# Sal003 alleviated intervertebral disc degeneration by inhibiting apoptosis and extracellular matrix degradation through suppressing endoplasmic reticulum stress pathway in rats

**DOI:** 10.3389/fphar.2023.1095307

**Published:** 2023-01-20

**Authors:** Yan Chen, Baixing Li, Yue Xu, Tangjun Zhou, Changqing Zhao, Jie Zhao

**Affiliations:** ^1^ Department of Orthopaedic Surgery, Shanghai Ninth People’s Hospital, Shanghai Jiao Tong University School of Medicine, Shanghai, China; ^2^ Shanghai Key Laboratory of Orthopaedic Implants, Shanghai Ninth People’s Hospital, Shanghai Jiao Tong University School of Medicine, Shanghai, China; ^3^ Changshu Hospital Affiliated to Nanjing University of Chinese Medicine, Suzhou, Jiangsu, China

**Keywords:** ER stress pathway, Sal003, intervertebral disc degeneration, apoptosis, extracellular matrix degradation

## Abstract

Apoptosis and extracellular matrix degradation of the nucleus pulposus are the main initiators of intervertebral disc degeneration (IVDD) and can be explained by endoplasmic reticulum (ER) stress. Thus, pharmacological therapy aimed at suppressing this pathway may be a promising approach for the management of intervertebral disc degeneration. In this study, we aimed to explore the protective effects of Sal003 against intervertebral disc degeneration and its underlying mechanisms. Thapsigargin (Tg)-stimulated rat nucleus pulposus cells and a needle puncture-induced intervertebral disc degeneration rat model were used to explore the protective effects of Sal003. Our results showed that Sal003 inhibited apoptosis and extracellular matrix degradation by suppressing the endoplasmic reticulum stress pathway. The therapeutic effects of Sal003 were also observed in the intervertebral disc degeneration rat model, as evidenced by improved degeneration along with decreased apoptosis and extracellular matrix degradation in intervertebral discs. Our results demonstrated Sal003 as a potential treatment for intervertebral disc degeneration.

## 1 Introduction

Low back pain (LBP) is one of the top ten causes of injury affecting disability-adjusted life-years (DALYs) (Diseases and Injuries 2020). It is also a common chief complaint among outpatients (Traeger et al., 2021). Furthermore, intervertebral disc degeneration (IVDD) is regarded as an important risk factor for LBP and is characterized by the death of nucleus pulposus (NP) cells and degradation of the extracellular matrix (ECM) (Kleimeyer et al., 1976; Livshits et al., 2011; Mohd Isa et al., 2022; Zhang et al., 2022a). However, the mechanisms underlying IVDD remain unclear and need to be further explored (Li et al., 2022a; Li et al., 2022b). Therefore, it is necessary to pay attention to the mechanism and treatment of IVDD (Song et al., 2021; Yin et al., 2021).

Endoplasmic reticulum (ER) stress is generally considered as a series of molecular and biochemical changes inside cells due to ER dyshomeostasis when cells are subjected to various stimuli (Luo et al., 2022). It mainly includes dysfunction of protein folding, impairment of protein transportation, and depletion of Ca^2+^ in the ER lumen (Groenendyk and Michalak 2005; Kadowaki et al., 2004; Zhao et al., 2010). ER stress is closely related to aging, apoptosis, and ECM degradation in the pathology of numerous diseases including osteoarthritis, Alzheimer’s disease, amyotrophic lateral sclerosis, and rheumatoid arthritis (Hosseinzadeh et al., 2016; Paschen and Mengesdorf 2005; Rahmati et al., 2018; Rellmann et al., 2021; Salminen et al., 2009). Our results and those of other groups have demonstrated that ER stress is a detrimental process when NP cells are subjected to stimuli, and repression of ER stress could mitigate IVDD (Lin et al., 2021; Luo et al., 2019; Wang et al., 2021). Thapsigargin (Tg), a classical inducer of ER stress *in vitro*, can increase cytoplasmic Ca^2+^ levels by suppressing cells from pumping Ca^2+^ back into the ER (Sikkeland et al., 2019; Zhang et al., 2014). In addition, Tg has been widely acknowledged as a commonly used activator of ER stress and ER stress-related changes in NP cells in *ex vivo* studies on IVDD (Krupkova et al., 2018; Novais et al., 2021; Yang et al., 2022; Zhang et al., 2022b).

Sal003 is a recently discovered small molecule that functions as a DNA damage-inducible protein (GADD34) inhibitor. It has also been demonstrated that Sal003 could modulate ER stress and alleviate ROS production in various tissues such as the cornea, kidney, and muscle but not in NP cells (Fujita et al., 2021; Ju et al., 2017; Soiberman et al., 2019). Here, we tested the hypothesis that Sal003 mitigates IVDD by modulating ER stress.

Based on these results, we explored whether Sal003 could exert protective effects on the Tg-induced abnormal phenotypes of NP cells. We also determined whether it could modulate ER stress. Furthermore, the potential therapeutic effects of Sal003 on IVDD were explored *in vivo*.

## 2 Materials and methods

### 2.1 Reagents

Sal003 was obtained from Selleck Chemicals (S7437, Houston, TX, United States). DMSO was used to dissolve Sal003. It was then stored at −20°C with a stock solution of 40 mM.

### 2.2 NP primary cells isolation and culture

Cervical dislocation was performed in six-week-old male Sprague-Dawley (SD) rats. The rats were then rinsed with 75% ethanol for 30 min. We obtained NP tissues from their intervertebral discs (Co1–Co6) and soaked them in 1% collagenase II for 2 h. The cells were cultured in Dulbecco’s Modified Eagle’s medium (DMEM) supplemented with 10% fetal bovin serum and 1% penicillin-streptomycin under routine conditions (37°C with 5% CO^2^), followed by centrifugation (37°C, 300 × *g* for 5 min) and suspension.

### 2.3 Cell counting Kit-8 assay

The Cell Counting Kit-8 Assay (CCK-8 kit, E606335, Sangon Biotech Co. Ltd., Shanghai, China) was used to measure cell toxicity and proliferation. To determine cell toxicity, cells were cultured in 96-well plates by 1*10⁴ before they were administered with different doses of Sal003 (0, 1.25, 2.5, 5, 10, 20, and 40 μM) for 24 h. To ascertain proliferation, the culture of cells was performed in 96-well plates at 2.5 × 10³ with previous concentrations and were administered Sal003 for 24, 48, and 72 h. Fresh complete media containing 10 μL of CCK-8 reagent was added into plates according to specific time periods, and the cells were incubated at 37°C for 2 h. Absorbance at 450 nm (optical density values, OD) was assessed using an Infinite M200 Pro reader (Tecan Life Sciences, Männedorf, Switzerland).

### 2.4 RNA extraction and real-time quantitative PCR analysis

NP primary cells were pre-administered Sal003 (5 μM) for 2 h following which Tg (1 μM) was used to stimulate cells for 24 h. Afterwards, extraction was performed using TRIzol reagent (Thermo Fisher Scientific, Waltham, MA, United States) according to the manufacturer’s instructions. Complementary DNA (cDNA) was obtained from RNAs using a cDNA Synthesis Kit. Real-time quantitive PCR (RT-qPCR) was performed using the TB Green Premix Ex Taq Kit (Takara Bio) in a Real-Time PCR System. NCBI BLAST was used to design specific primer pairs. Primer information is provided in Table 1. The target gene expression was adjusted and normalized to that of β-actin. Relative gene expression levels were quantified using the comparative 2^−ΔΔCT^ method.

**TABLE 1:** Primers information.

Target gene	Accession number	Primer sequences 5′→3′
β-actin	NM_031144.3	F:GTCCACCCGCGAGTACAAC
		R:GGATGCCTCTCTTGCTCTGG
MMP3	NM_133523.3	F:TTTGGCCGTCTCTTCCATCC
		R:GCATCGATCTTCTGGACGGT
MMP9	NM_031055.2	F:TCTGCCTGCACCACTAAAGG
		R:CAGGCTGTACCCTTGGTCTG
MMP13	NM_133530.1	F:TGCTGCATACGAGCATCCAT
		R:TGTCCTCAAAGTGAACCGCA
ADAMTS5	NM_198761.2	F:CGACAAGAGTCTGGAGGTGAG
		R:CGTGAGCCACAGTGAAAGC
Aggrecan	XM_039101035.1	F:TCCAAACCAACCCGACAAT
		R:TCTCATAGCGATCTTTCTTCTGC
ATF4	NM_024403.2	F:GACCGAGATGAGCTTCCTGAACAG
		R:CCGCCTTGTCGCTGGAGAAC
ATF6	NM_001107196.1	F:CCAGAAGCACGGGTTCAGAT
		R:GCAGGGCTCACACTAGGTTT
BiP	NM_023083.2	F:ACACCTGACCGACCGCTGAG
		R:GCCAACCACCGTGCCTACATC
CHOP	NM_001109986.1	F:CTGAAGAGAACGAGCGGCTCAAG
		R:GACAGGAGGTGATGCCAACAGTTC
TRAF2	NM_001107815.2	F:CGAAGACCGTTGGGGCTTT
		R:TCGTGGCAGCTCTCGTATTC

### 2.5 Protein extraction and western blot analysis

To determine changes in the matrix metalloproteinase (MMP) family, apoptosis, and long-term activated signaling at the protein level, primary NP cells were treated as described previously. After washing twice with 1 × phosphate-buffered saline (PBS), RIPA mixed with phosphatase and protease inhibitors (Roche, Basel, Switzerland) was used for the extraction of total cellular proteins. The proteins in the supernatant were collected for further quantification after centrifugation at 12,000×*g* for 15 min using a BCA protein quantification kit (23,227, Thermo Fisher Scientific, Waltham, MA, United States). After dissolving them in SDS-sample loading buffer, the proteins (approximately 25 μg) were separated on 4%–20% SDS-PAGE gels and electroblotted onto 0.22-μm PVDF membranes (Merck-Millipore, CA, United States). Membranes were blocked with 5% skim milk at room temperature (RT) for 2 h and then incubated with primary antibodies (diluted 1:1,000 in 5% BSA–TBST) overnight at 4°C. Primary antibodies against MMP3 (ab52915), MMP9 (ab228402), MMP13 (ab39012), aggrecan (ab3773), Bax (ab32503), and Bcl-2 (ab196495) were purchased from Abcam (Cambridge, United Kingdom). Other antibodies used, such as β-actin (D6A8), Cleaved Caspase-3 (ASP175), Bip (3183S), CHOP (L63F7), XBP-1s (E9V3E), and IRE1α (14C10) were purchased from Cell Signaling Technology (Danvers, MA, United States). Membranes were washed three times with TBST for 10 min and then incubated with anti-rabbit or anti-mouse secondary antibodies (1:10,000 dilution) for 1 h with protection from light exposure. After washing three times with TBST, the protein immunoreactivity of the membranes was analyzed using a fluorescence imaging system (LI-COR Biosciences, Lincoln, NE, United States). Measurements of OD values were performed using ImageJ software (National Institutes of Health, United States).

### 2.6 Immunofluorescence

For immunofluorescence detection, slides were used to seed cells in a six-well plate at a density of 4×10^5^ cells per well for 8 h, waiting for attachment and proliferation. NP cells were pre-administered with or without Sal003 (5 μM) for 2 h and subsequently treated with Tg (1 μM) for 24 h. NP cells were fixed with paraformaldehyde (4%) for 15 min at RT. For permeation of the membranes, 0.25% Triton X-100 was used for 10 min and then washed three times with PBS. After 2 h of blocking with 5% BSA at RT, cells were incubated with primary antibodies against MMP13, Aggrecan, CHOP, XBP-1s (diluted 1:200, Abcam) overnight at 4°C. PBS was used to wash the cells thrice the next day. Alexa Fluor 488 or 594 secondary antibody (anti-rabbit, Cell Signaling Technology) was used for incubation of the cells for 1 h after which they were washed three times. After incubation for 15 min with DAPI and washing, the images were obtained using a fluorescence microscope (Leica SP8 confocal microscope, Germany).

### 2.7 High density culture

Ten μL NP cells at a density of 1.0×10^7^ cells/mL were seeded in 24-well plate. After 2 h of cell attachment, each well in plate was added DMEM medium with 2.5% FBS and 1% insulin transferrin selenium (ITS) (Gibco, Thermo Fisher Scientific, Waltham, MA, United States) with Tg and Sal003 as mentioned before. The medium was changed every 2 days. The cluster of NP cells was subjected to alcian blue and toluidine blue dye overnight at room temperature following fixation by 4% PFA for 10 min to assess the production of collagen synthesis after culture for 7 days.

### 2.8 Flow cytometry assay

Apoptotic NP cells were determined using the Annexin V 633 Apoptosis Detection Kit (AD11, Dojindo, Kumamoto, Japan), according to the manufacturer’s protocol. The apoptotic rate (%) in various groups was examined using flow cytometry (BD Co., United States).

### 2.9 Surgical procedure

Twenty male SD rats aged 12 weeks were kept under appropriate circumstances preoperatively and postoperatively. Percutaneous needle puncture was used to construct an IVDD model in rats (Han et al., 1976). Briefly, anesthesia was administered to the rats by intraperitoneal injection of pentobarbital sodium (40 mg/kg of body weight), and 75% ethanol was used to disinfect the tails. A 20G sterile needle was used to puncture the Co7/8 and Co8/9 discs from the dorsal skin into the NP center. The IVDD model was completed by a subsequent rotation of 360° and holding for 30 s in position. Eventually, re-sterilization was performed after pulling out the needle to avoid infection. 5 μL Sal003 of 5 µM was given to the Co8/9 discs every week *via* a microsyringe as IVDD + Sal003. The IVDD group was administered the same volume of saline weekly into the Co7/8 discs. Co6/7 discs were not administered any treatment as they were the control group.

### 2.10 X-ray analysis

The rats underwent X-ray examination 4 weeks postoperatively for evaluation of the intervertebral space. Digital imaging was captured in the anteroposterior axis using 21 lp/mm detectors supplied with ×5 geometric magnification (Faxitron VersaVision; Faxitron Bioptics LLC, Tucson, AZ, United States). Calculation of disc height indices (DHIs) was in accordance with a previous report in which the average IVD height (DHI) was calculated by averaging the measurements obtained from the anterior, middle, and posterior portions of the IVD and dividing it by the average of adjacent vertebral body heights. Changes in the DHI of injected discs were expressed as DHI% and normalized to the measured preoperative IVD height (DHI = 2× (D + E + F)/(A + B + C + G + H + I)% = postoperative DHI/preoperative DHI × 100) (Han et al., 2008).

### 2.11 Magnetic resonance imaging (MRI) analysis

IVDD progression was assessed by MRI. Four weeks postoperatively, the rats were fixed after anesthesia, and the signals of the assigned IVD section were acquired in T2-weighted images (T2WI) of the sagittal planes using a 1.5 T magnetic resonance scanner (Philips Eclipse, Cleveland, OH, United States). Subsequently, a grading system for determining IVDD levels based on MRI scan was measured by Pfirrmann grades: A homogeneous bright white structure as Grade I, inhomogeneous white structure and possible horizontal bands as Grade II, clear distinction between annulus and nucleus as Grade III, no collapsed disc space as Grade IV, and collapsed disc space as Grade V (Masuda et al., 1976).

### 2.12 Histology analysis

Four% paraformaldehyde was collected and fixed in four coccygeal IVD tissues for at least 48 h. Then, paraffin was embedded in the samples, and 5 µm sections were obtained in the region of the midsagittal plane. Subsequently, sections were stained with hematoxylin and eosin (HE), alcian blue (AB), toluidine blue (TB) and safranin O-fast green (SO-FG) to assess the IVD structures. The histological score was calculated as previously described (Ji et al., 2018).

### 2.13 Immunohistochemistry

Deparaffinization and rehydration were first administered to sections, and 3% hydrogen peroxide (H_2_O_2_) was used for 20 min to block endogenous peroxidase. The primary antibodies were incubated overnight at 4°C, followed by blocking with 10% goat serum for 2 h at RT. Sections were then washed three times with PBS and incubated with HRP-conjugated secondary antibody for 2 h at RT the next day. Digital images were obtained using a Leica DM4000 B microscope (Leica Microsystems). The integrated optical density (IOD) was measured for semi-quantitative analysis using Image Pro Plus software (version 6.0; Media Cybernetics, Silver Spring, MD, United States).

### 2.14 Tunel

Apoptosis of NP cells was examined by terminal deoxynucleotidyl transferase dUTP nick end labeling (TUNEL) using a TUNEL Kit (C1086, Beyotime Biotechnology Co., Shanghai, China). Quantification of positive cells was performed in three randomly selected fields from each sample using Image-Pro Plus 6.0 (Media Cybernetics, Silver Spring, MD, United States). DAPI staining was used to estimate the total cell number under a fluorescence microscope.

### 2.15 Statistical analysis

The experiments in our study were performed at least three times to obtain data, which are presented as means ± standard deviations. Differences between two groups were assessed by the *t*-test, while significant differences among groups were assessed by ANOVA. Statistical significance was set at *p* < 0.05. GraphPad Prism 8.3 (GraphPad Software, San Diego, CA, United States) was utilized for data analysis and creating relevant figures.

## 3 Results

### 3.1 Influences of Sal003 on viability of NP cells

**FIGURE 1:**
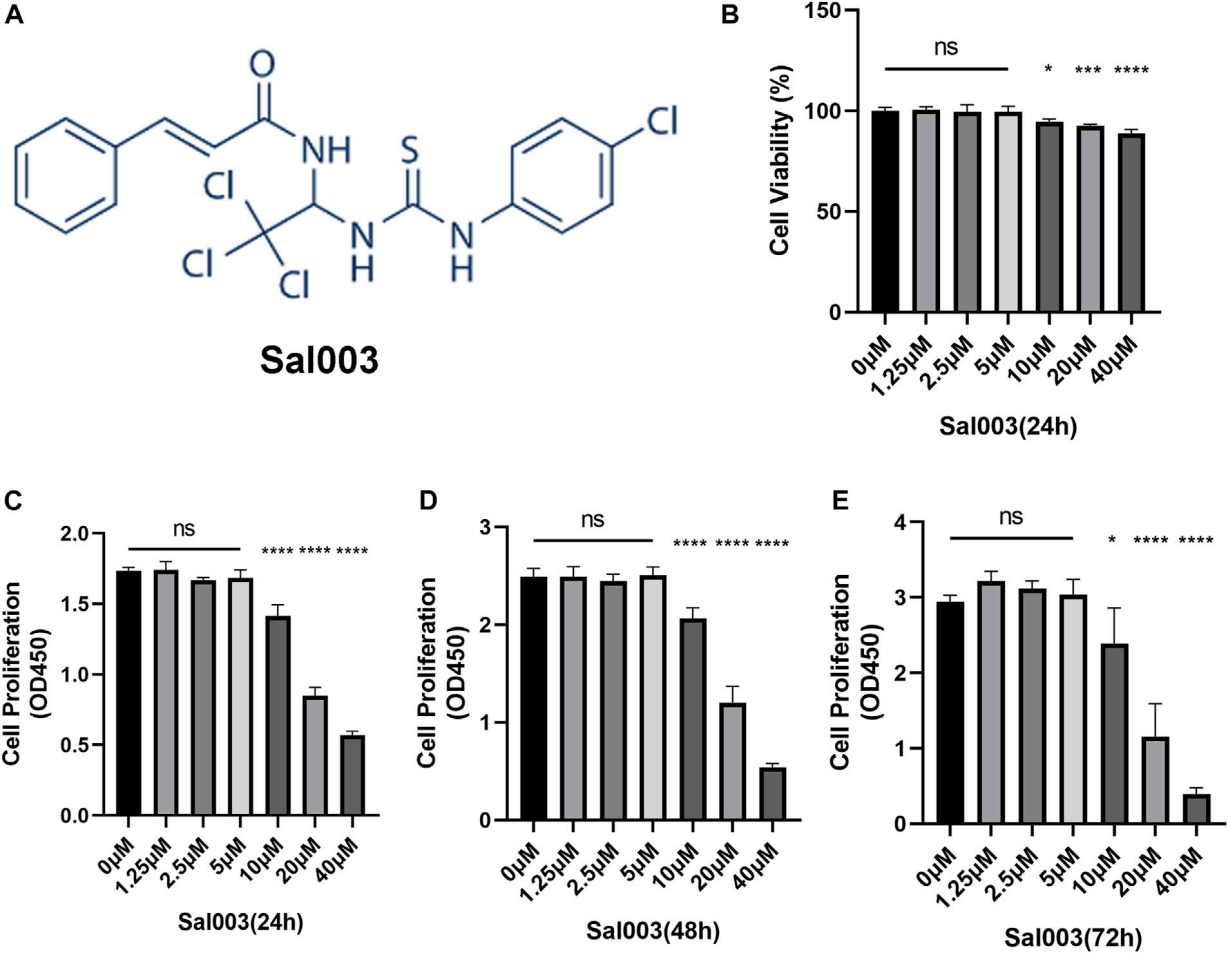
The chemical structure of Sal003 and effects of Sal003 on NP cells viability and proliferation. **(A)** The chemical structure of Sal003. **(B)** NP cells at a density of 1×10^4^ cells/well were treated with Sal003 at different concentrations for 24 h. CCK-8 experiments were carried out to evaluate the cytotoxicity. **(C–E)** The effects of Sal003 on proliferation was also determined by CCK-8 experiments. NP cells at a density of 2.5×10^3^ cells/well were incubated with Sal003 for 24 h **(C)**, 48 h **(D)** or 72 h **(E)**. All data are presented as mean ± SD. *p < 0.05, **p < 0.01, ***p < 0.001, ****p < 0.0001, *n* = 3.

Sal003 is presented in Figure 1A.

To analyze cytotoxicity, each well of the 96-well plates was used for seeding 10,000 NP cells and cultivated for 24 h at concentrations of 0, 2.5, 5, 10, 20, and 40 μM Sal003. The cytotoxicity of Sal003 was calculated based on the 0 μM Sal003 group. Sal003 exhibited cytotoxicity in NP cells at concentrations of 10 μM or higher (Figure 1B).

To determine cell proliferation, each well of the 96-well plates was seeded by 2,500 NP cells and cultured for 24 h at concentrations of 0, 2.5, 5, 10, 20, and 40 μM Sal003. To ensure the proliferation of Sal003, the cells were subjected to a CCK-8 assay for a time period ranging from 24 to 72 h following seeding. Sal003 showed an inhibitory influence on the proliferation of NP cells at 20 μM or more. NP cells treated with 5 μM Sal003 were significantly higher than those treated with other concentrations at 48 and 72 h (Figures 1C–E). Thus, 5 μM Sal003 was used in the following experiments.

### 3.2 Sal003 mitigated ECM degradation induced by Tg *in vitro*

**FIGURE 2:**
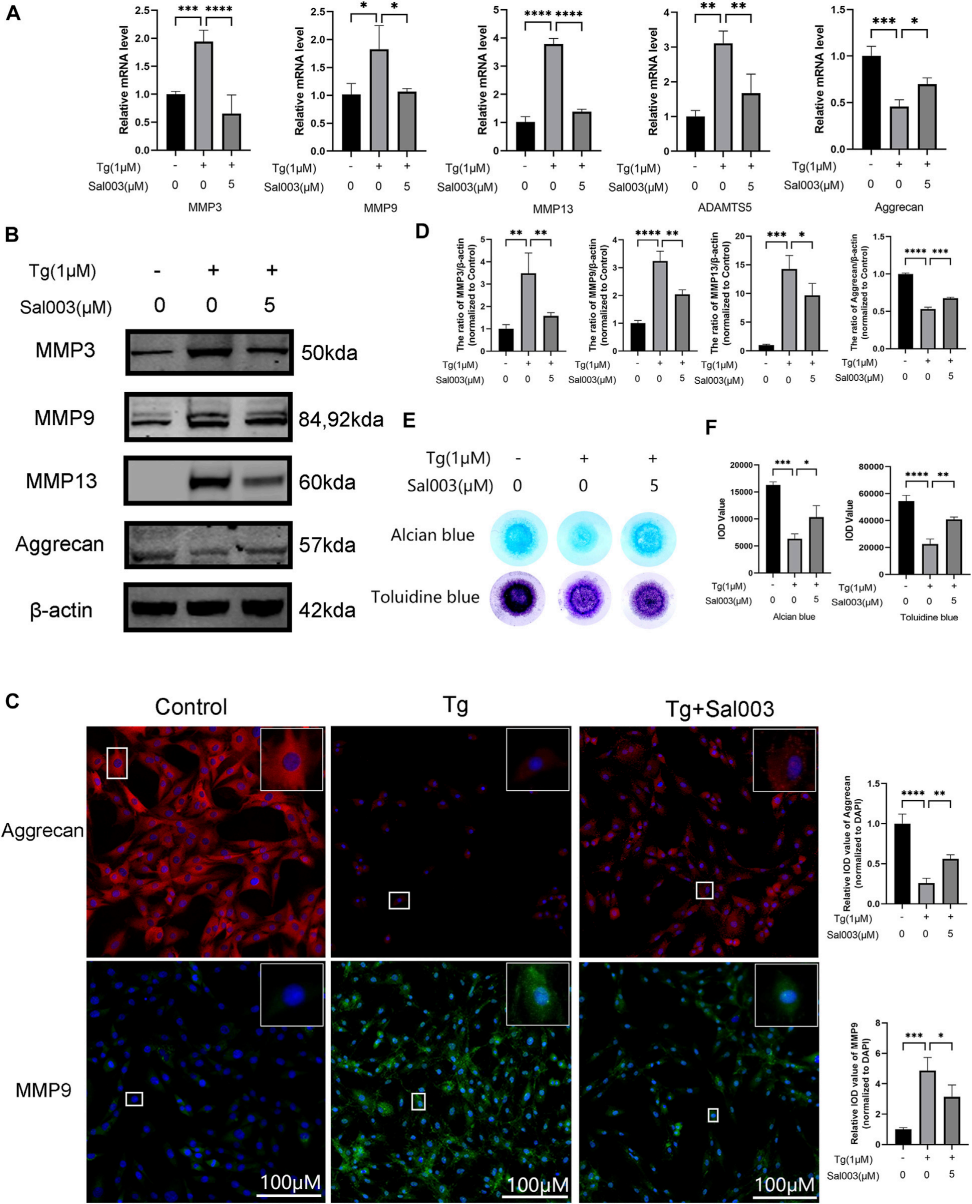
Sal003 prevented NP cells from extracellular degradation induced by Tg. **(A)** The relative mRNA expression of MMP3, MMP9, MMP13, ADAMTS5, and Aggrecan were analyzed by RT-qPCR. **(B,D)** The expression of MMP3, MMP9, MMP13, and Aggrecan at protein level were detected by Western Blotting. **(C)** The representative merged images of MMP9 and Aggrecan were obtained by immunofluorescence combined with DAPI staining for nuclei (scale bar: 100 µm). Values of IOD were measured to determine the results of immunofluorescence. **(E,F)** Alcian blue and toluidine blue staining of high-density culture of NP cells and quantitative analysis. All data are presented as mean ± SD. *p < 0.05, **p < 0.01, ***p < 0.001 and ****p < 0.0001, *n* = 3.

RT-qPCR, Western Blotting, and immunofluorescence were performed to determine the changes in the ECM. RT-qPCR showed upregulation of MMP3, MMP9, MMP13, and ADAMTS5, which are related to the increase in catabolic levels following stimulation by Tg. Furthermore, aggrecan (considered one of the dominant factors of ECM) levels decreased after treatment with Tg. These Tg-induced harmful factors were partially reversed by Sal003 treatment (Figure 2A).

In addition, Western Blotting demonstrated that ECM degradation, including a decrease in aggrecan and an increase in MMP3, MMP9, and MMP13 protein levels, induced by Tg, could be reversed by Sal003 (Figures 2B, D). The immunofluorescence staining results for MMP13 and aggrecan were also in accordance with the results mentioned in the study (Figure 2C). Micromass culture results were in accordance with previous outcome (Figures 2E, F).

### 3.3 Sal003 alleviated Tg-triggered apoptosis *in vitro*

**FIGURE 3:**
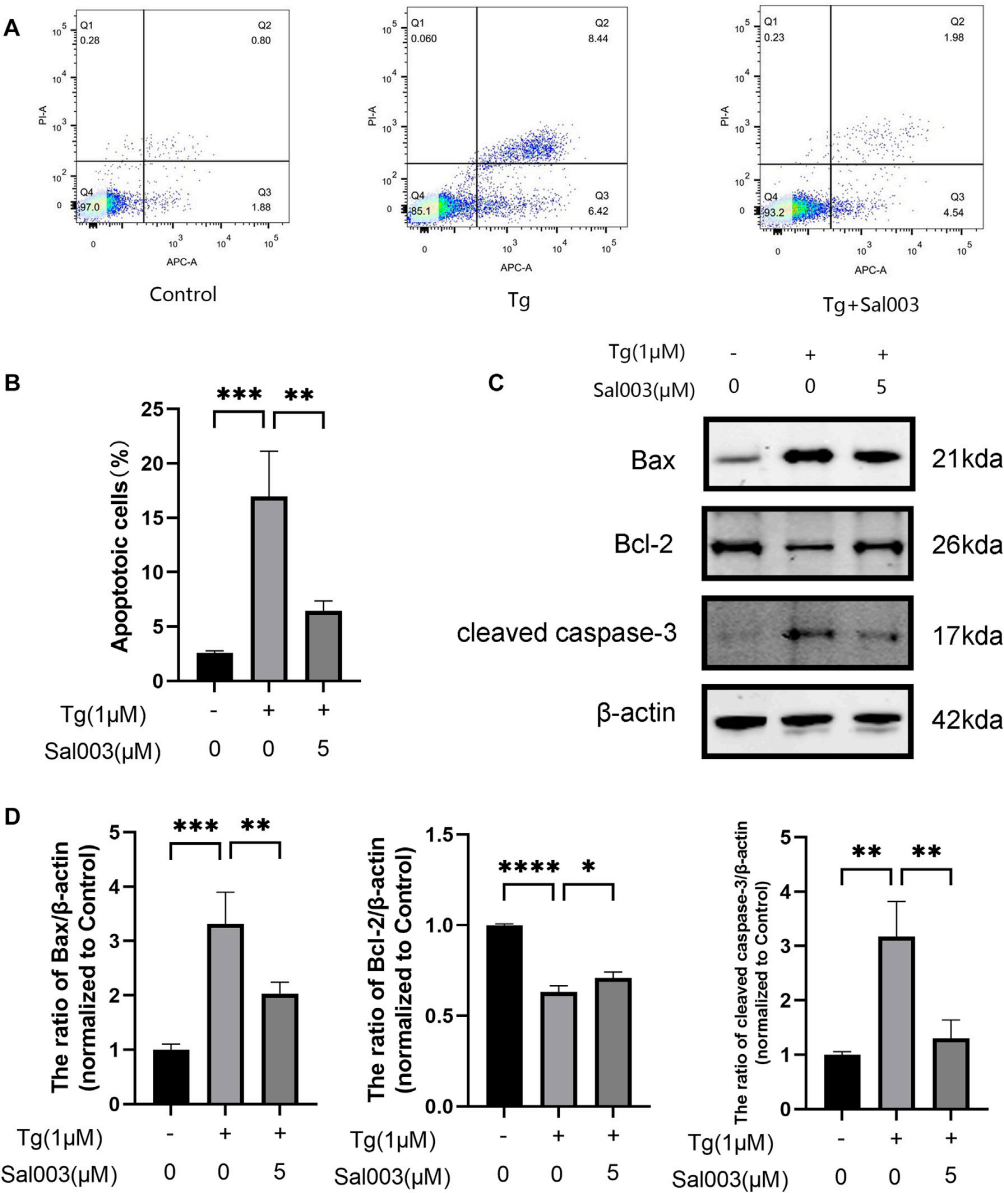
Sal003 mitigated Tg-induced apoptosis of NP cells. **(A,B)** Stained with Annexin V-633 and PI, apoptotic NP cells rates in different groups were determined by flow cytometry. **(C,D)** The expression of Bax, Bcl-2 and cleaved caspase-3 at the protein level were determined by western blotting. All data are presented as mean ± SD. *p < 0.05, **p < 0.01 and ***p < 0.001, ****p < 0.0001, *n* = 3.

To determine the potential protective effects of Sal003 in Tg-stimulated NP cells, flow cytometry and Western Blotting were performed to evaluate apoptotic activity. The results of flow cytometry indicated that the number of apoptotic NP cells in the Tg group was markedly higher than that in the Sal003 pretreated group (Figures 3A, B). Moreover, apoptosis-related proteins were detected using Western Blotting analysis (Figures 3C, D). The results indicated that Sal003 increased Bcl-2 and inhibited Bax and cleaved caspase-3 in NP cells. Our results suggest that ER stress-related apoptosis of NP cells caused by Tg induction is decreased following pretreatment with Sal003.

### 3.4 Sal003 reversed Tg-triggered ER stress *in vitro*

**FIGURE 4:**
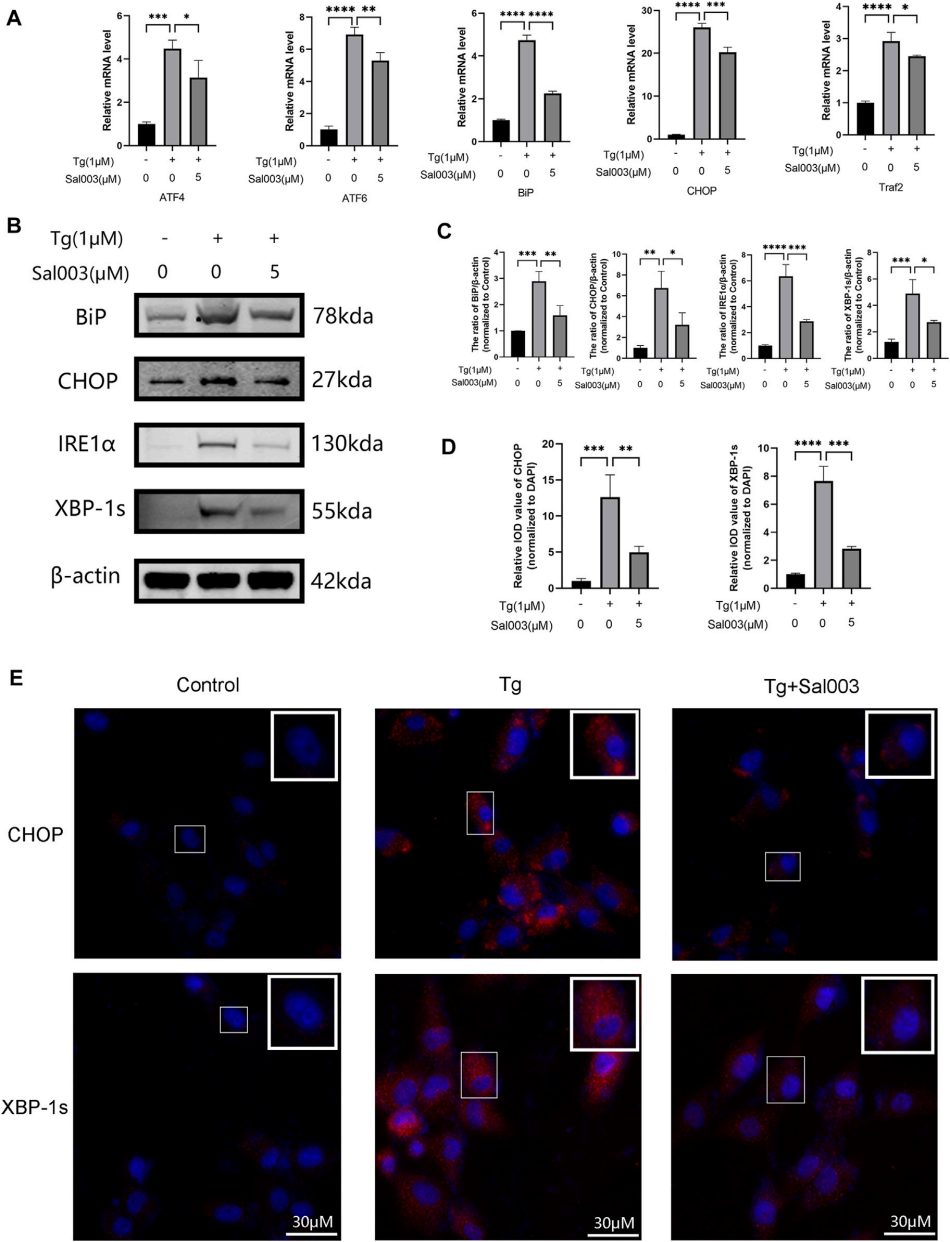
Sal003 downregulated Tg-induced ER stress in NP cells. **(A)** The relative mRNA expression of ATF4, ATF6, BiP, CHOP, and Traf2 were analyzed by RT-qPCR. **(B,C)** The protein level expression of BiP, CHOP, IRE1α and XBP-1s treated by Sal003 were measured by western blotting. **(D,E)** The representative merged images of CHOP and XBP-1s were obtained by immunofluorescence combined with DAPI staining for nuclei (scale bar: 30 µm). Values of IOD were measured to determine the results of immunofluorescence. All data are presented as mean ± SD. *p < 0.05, **p < 0.01, ***p < 0.001 and ****p < 0.0001, *n* = 3.

Using RT-PCR and Western Blotting, NP cells were treated with Tg to examine whether Sal003 exerted cytoprotective effects under ER stress. ATF4, ATF6, Bip, and CHOP levels were markedly higher in the Tg-treated group than those in the control group, and Sal003 partially but significantly reversed these increases (Figure 4A). In addition, Western Blotting showed that Sal003 inhibited Tg-activated ER stress by decreasing Bip, CHOP, XBP-1s, and IRE1α at the protein level (Figures 4B, C). The immunofluorescence staining results for CHOP and XBP-1s were also in accordance with the results mentioned in the study (Figures 4D, E).

### 3.5 Sal003 rescued IVDD induced *via* needle puncture in the rat tail *in vivo*

**FIGURE 5:**
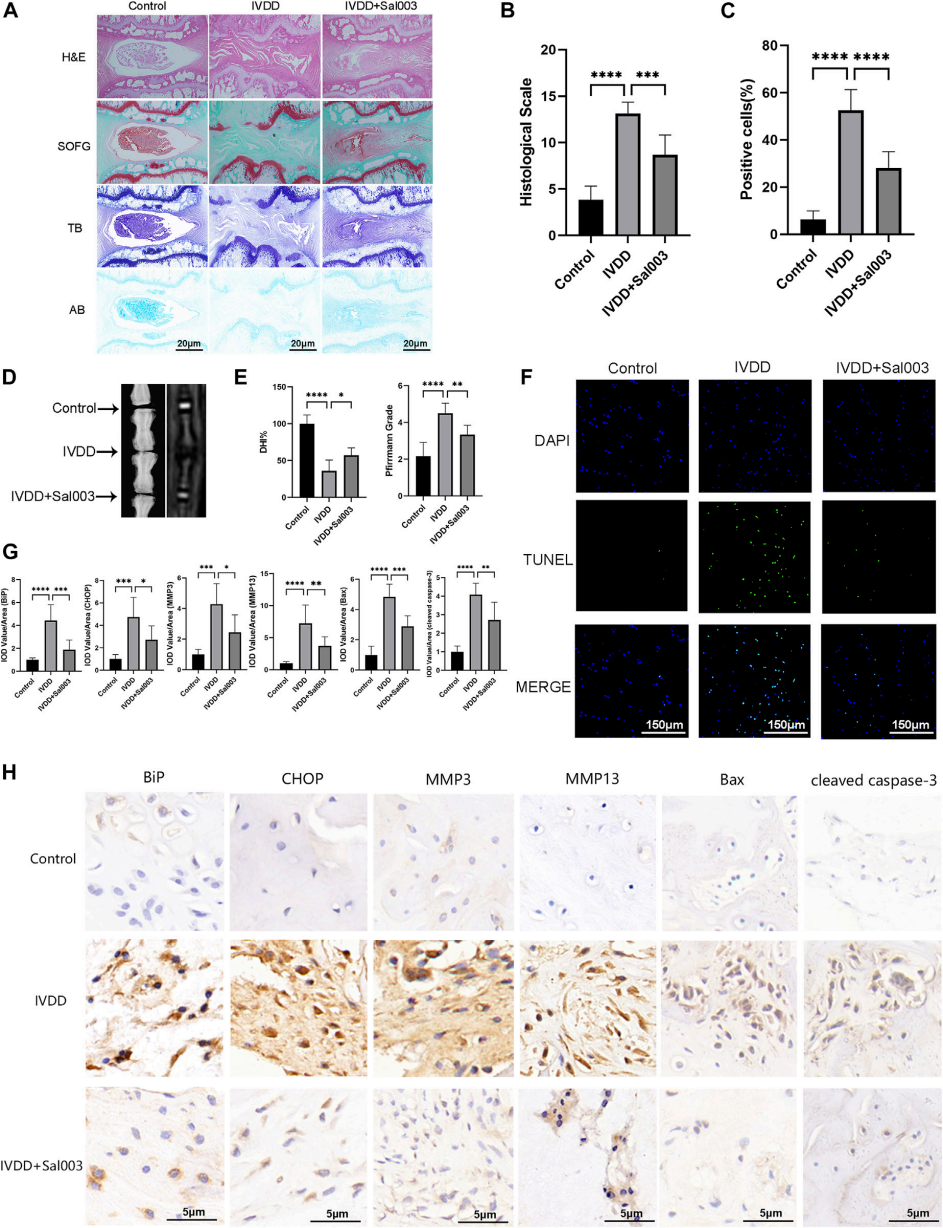
Sal003 ameliorated IVDD in a rat puncture-induced model *in vivo*. **(A,B)** The representative images and histological scales of HE, SO-FG, AB and TB staining of punctured IVD in different groups (scale bar: 20 µm). **(C,F)** The semi-quantitative analysis and representative images of TUNEL staining for IVD tissues in different groups (scale bar: 150 µm). **(D,E)** The representative X-ray and MRI images of a rat tail after 4 weeks of surgery with the disc height index and Pfirrmann grades shown in different groups at 4 weeks. **(G)** The IOD value/area was calculated to assess the results of immunohistochemistry staining through semi-quantitative analysis. **(H)** The expression of BiP, CHOP, MMP3, MMP13, Bax and cleaved caspase-3 at IVD tissues in different groups were determined by immunohistochemistry staining (scale bar: 5 µm). All data are presented as mean ± SD. *<0.05. **p < 0.01, ***p < 0.001 and ****p < 0.0001, *n* = 6.

Experiments were performed to determine whether Sal003 could function as a therapeutic agent for IVDD *in vivo*. The rat IVDD model was established by needle puncture of caudal discs. Radiography and MRI were performed 4 weeks postoperatively. HE, SOFG, AB, and TB staining were also used to assess morphological differences in IVD following Sal003 administration. After assessing histomorphology, observed findings included contraction, reduction of NP cells, disorganization at the border between the nucleus pulposus and annulus fibrosus, and extensive loss of proteoglycans in the IVDD group. However, Sal003 treatment partially mitigated these detrimental changes at the histopathological levels, demonstrating slight shrinking and loss of NP cells and proteoglycan. Histological score analysis also demonstrated the impact of Sal003 in delaying IVDD progression (Figures 5A, B). As shown in Figures 5D, E, the IVDD group showed decreased disc signal and intervertebral height after surgery. However, Sal003 partially reversed the collapsed disc height and decreased the MRI signal. TUNEL assay was also applied to determine whether Sal003 could mitigate apoptosis *in vivo*. Consistent with our *in vitro* results, Sal003 inhibited apoptosis during IVDD (Figures 5C, F). Furthermore, immunohistochemical staining of BiP, CHOP, MMP3, MMP13, Bax and cleaved caspase-3 demonstrated the mitigation by Sal003 of ECM degradation, ER stress activation and apoptosis in the rat IVDD model, which was also in agreement with the results of our *ex vivo* experiments (Figures 5G, H).

## 4 Discussion

**FIGURE 6:**
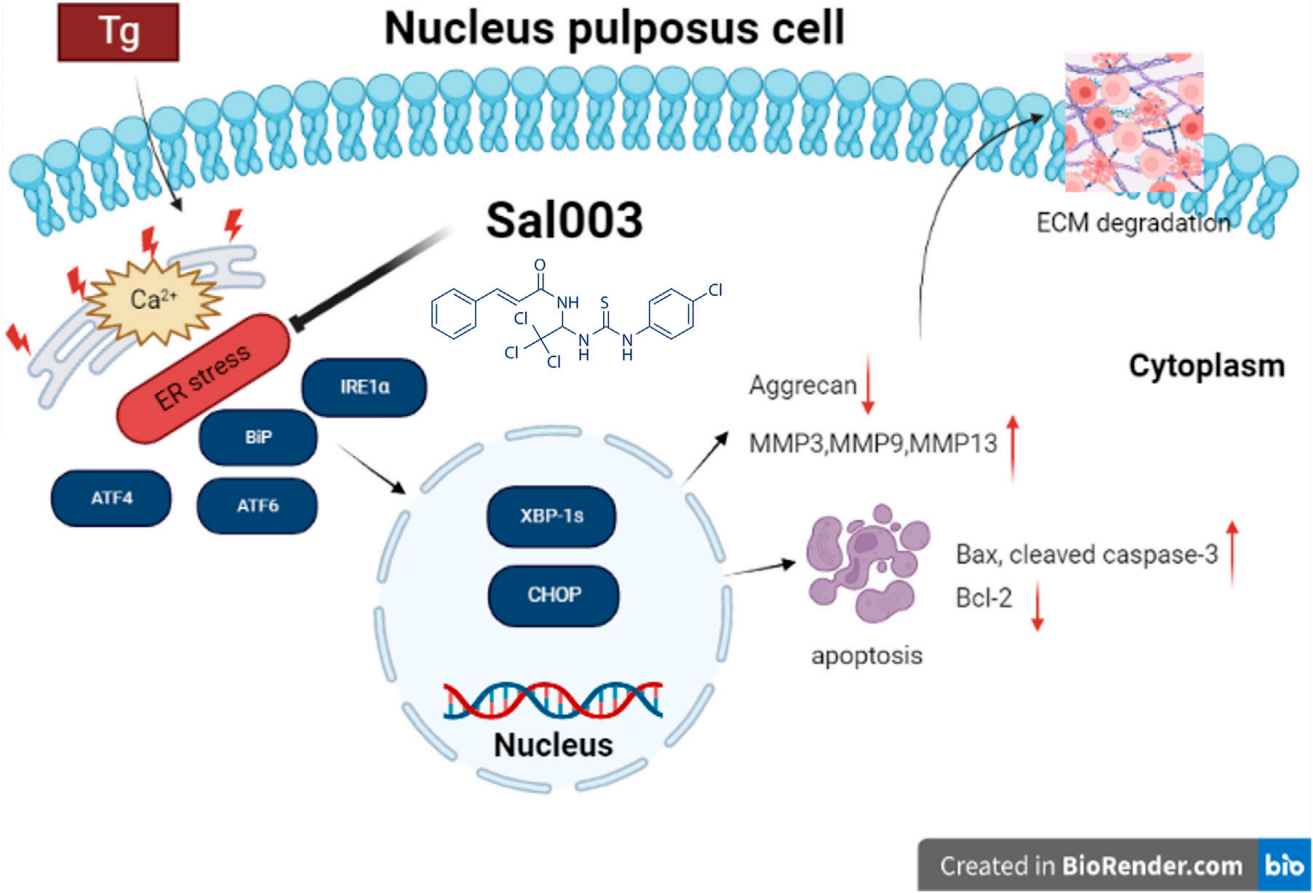
Potential molecular mechanisms involved in therapeutic impacts of Sal003 on IVDD. Created with BioRender.com.

IVDD is a degenerative joint disease characterized by excessive apoptosis of NP cells and degradation of ECM (Liang et al., 2022). ER stress is a potential mechanism underlying IVDD. In our study, Tg induced ER stress by inhibiting the Ca^2+^-ATPase activity. Sal003 is a small-molecule compound that exerts a regulatory effect under ER stress (Dadey et al., 2018; Darini et al., 2019). Studies have reported that Sal003 promotes cell expansion and reduces DNA damage (Fujita et al., 2021; Soiberman et al., 2019). In this study, we discuss the relationship between the protective effect of Sal003 and ER stress both *in vitro* and *in vivo* for the first time. Our results indicated that Sal003 could regulate the ER stress pathway when Tg was administered to rat NP cells, thus showing the characteristics of inhibiting apoptosis and reducing matrix degradation. Our *in vivo* results showed for the first time that Sal003 could significantly inhibit ER stress and reduce NP cell apoptosis and ECM degradation. In general, our study demonstrated that Sal003 could exert therapeutic effects on IVDD, both *in vivo* and *in vitro* (Figure 6).

Excessive ER stress can lead to apoptosis and extracellular matrix (ECM) degradation. ER stress-induced apoptosis involves transcriptional activation of the CHOP pathway, activation of BiP, and induction of the caspase pathway (Puthalakath et al., 2007). In human IVDD, the expression of ER stress-related biomarkers, including p-PERK, ATF4, BiP, and CHOP, in NP is positively correlated with degeneration grade (Fujii et al., 2018; Liao et al., 2019; Lin et al., 2021). Animal experiments have further proven that the expression of ER stress-related markers is related to the degeneration grade and senescence of rat intervertebral discs (Xie et al., 2017). Previous studies showed that the degree of apoptosis in intervertebral discs was lower than that of the control group after CHOP gene knockout, and MRI signal and histological scores were significantly improved after injection of CHOP shRNA into rats (Zhang et al., 2011). Overall, prolonged activation of IRE1α and CHOP can trigger apoptosis under physiological and pathophysiological conditions (Szegezdi et al., 2006). Unfolded protein reaction (UPR)-induced apoptosis may serve as a useful method to eliminate uncorrected cells under physiological ER stress (Tabas and Ron 2011). However, chronic ER stress can also induce pathological apoptosis (Kaufman 2002). We found that Sal003 inhibited Tg-induced activation of ER stress pathways, including IRE1α and CHOP, thus exerting anti-apoptotic functions by regulating the expression of Bax/Bcl-2, which was also in accordance with a previous report (Iurlaro and Munoz-Pinedo 2016). Furthermore, ER stress can also affect the synthesis and secretion of ECM proteins, causing protein aggregates to accumulate in the ER. Cells secreting ECM would die *via* apoptosis, necroptosis, and autophagy, which could disrupt homeostasis (Rellmann et al., 2021). In our study, we also explored whether the protective effects of Sal003 were related to attenuation of ER stress. RT-qPCR and Western blotting analysis showed that Sal003 inhibited Tg-induced apoptosis of NP cells and ECM degradation by regulating ER stress signaling pathways. The downregulation of ATF4, CHOP, BiP, IRE1α, XBP-1s, cleaved caspase-3, Bax, and MMP families, as well as the upregulation of Aggrecan and Bcl-2 proved this point. Our results showed that Sal003 reduced the apoptosis of NP cells and mitigated ECM degradation by reducing ER stress.

Some studies have shown that Sal003 can regulate various cell phenotypes by modulating ER stress (Table 2). In addition, Sal003 reduced the apoptosis of human renal proximal tubular cells by inhibiting p53 activation and reducing oxidative stress, according to a previously reported study (Ju et al., 2017). A recent study on musculoskeletal diseases demonstrated that Sal003 could upregulate p-eIF2α *in vitro* to further increase the expansion of muscle satellite cells (Fujita et al., 2021). However, it remains unclear whether Sal003 protects against IVDD by regulating the ER stress-related pathway. Based on this, we determined the effects of Sal003 on ER stress by measuring stress-related markers at the RNA and protein levels. Results showed that Sal003 could significantly inhibit ER stress pathway by downregulating ATF4, ATF6, BIP, CHOP, IRE1α and XBP-1s induced by Tg at the RNA and protein levels *in vitro*. Although the effects of Sal003 on ER stress showed minor differences from previous reports, it could be due to the interaction with other possible pathways and the specificity of NP cells based on the unique microenvironment of insufficient blood supply and hypoxia inside the intervertebral disc. Furthermore, cell fate after treatment with Sal003 could be determined by cell viability administered by increasing the concentration of Sal003 and differences in the pathological mechanisms of various diseases such as the discrepancy between tumor and musculoskeletal disorders (Table 2). The concentration of Sal003 in the *in vivo* experiment, which was 5μm, was determined according to our previous CCK-8 results. The drug dosage of 5 μL in this experiment was based on the volume of the IVD and previous reports (Yang et al., 2021; Zhou et al., 2022). Furthermore, the weekly injection of Sal003 was based on previous reports as well as the microenvironment inside IVD which is characterized by blood supply that is insufficient to maintain the continuity of the treatment (Li et al., 2022c; Ma et al., 2022). In addition, Sal003 significantly reduced the apoptosis of NP cells and degradation of ECM in puncture-induced IVDD rats and alleviated the expression of catabolic and stress-related markers *in vivo*. These results indicate that Sal003 reduced apoptosis and ECM degradation induced by ER stress in rat NP cells. The limitations of our study were that we failed to mention the protective effects of Sal003 on stress-related apoptosis and ECM degradation in human NP cells and the possible interactions between Sal003 and other pathways. These questions are the main direction for future research.

**TABLE 2:** Literature review of Sal003.

Experiment Type (or cell type) Treatment	Concentration	Measured parameters	Results	PMID
*In vivo*	20 μM	Inhibition of translation in NAc MSNs and eIF2α dephosphorylation	reduced cue-induced cocaine craving	29431650
Cue-induced seeking test	/0.5μL/hemisphere			
Intracranial injections				
*In vitro* experiment	10 μM	Promote eIF2α phosphorylation and caspase-7 activation	Enhanced apoptotic signaling	22354021
Hela cells				
*In vitro*	10 μM	Upregulate CHOP and downregulate cell viability	Reduced proliferation of GBM	29991528
Glioblastoma multiforme (GBM)				
Ionizing radiation				
*In vitro* experiment	5 μM	Stimulate P-eIF2α	Inhibition of colony forming	31086176
Her2+,BT474				
*In vitro* experiment	0.6, 1.2, 2.5, 5, 10 μM	Upregulation of ATF4, CHOP and p-eIF-2α	Stimulated integrated stress response (ISR)	35802384
Corneal Epithelial Cell				
*In vivo* experiment	50 μg/mL	Activation of ATF4 and Reduction of Col1a1	Decreased keratocyte density without affecting normal growth or development	34370978
Administration of ocular surface				
*In vivo* experiment	0, 2, 6, and 20 μM/side	Activation of ATF4 and eIF2α	Disrupted the reconsolidation of morphine- or cocaine-induced conditioned place preference (CPP)	25057203
Intra-basolateral amygdala (BLA) infusion				
*In vitro* experiment	0.625, 1.25, 2.5, 5, 10 μM	Decrease of hydroxyproline and COL1A1 transcription and eventual apoptosis in normal fibroblasts	Stimulated the ISR	31390655
Human corneal stromal cells				
*In vivo* and *ex vivo* experiment	10, 20 μM	Activation of p-eIF2α and ATF4	Intrahippocampal injection of Sal003 impairs contextual memory	17418795
Injection into the hippocampus				
Mouse embryonic fibroblasts				
*In vitro* experiment	5 μM	Activation of ATF4, p-eiF2α and HO-1	Inhibition of apoptosis and attenuation of oxidative stress	29039478
human renal proximal tubular cells, HK2 cells		Downregulation of P53, cleaved caspase-3, cleaved PARP and ROS		
*In vitro* experiment	10 μM	Maintain P-eIF2α	Permit the expansion of satellite cells *ex vivo*	33318147
Muscle satellite cells				

## 5 Conclusion

In conclusion, we demonstrated that Sal003 prevents apoptosis and ECM degradation by inhibiting the ER stress pathway in Tg-stimulated rat NP cells. Our *in vivo* experiment using an IVDD rat model confirmed that Sal003 could alleviate the degree of IVDD, which is related to the improvement of ER stress. These results indicate that Sal003 could be considered as a potential therapeutic treatment for IVDD.

## Data Availability

The raw data supporting the conclusion of this article will be made available by the authors, without undue reservation.
